# Loneliness and Optimism among Polish Nursing Students during the COVID-19 Pandemic: The Mediatory Role of Self-Efficacy

**DOI:** 10.3390/healthcare10060971

**Published:** 2022-05-24

**Authors:** Ewa Kupcewicz, Kamila Rachubińska, Aleksandra Gaworska-Krzemińska, Anna Andruszkiewicz, Ilona Kuźmicz, Dorota Kozieł, Elżbieta Grochans

**Affiliations:** 1 Department of Nursing, Collegium Medicum, University of Warmia and Mazury in Olsztyn, 14 C Zolnierska Street, 10-719 Olsztyn, Poland; ekupcewicz@wp.pl; 2 Department of Nursing, Pomeranian Medical University in Szczecin, 48 Zolnierska Street, 71-210 Szczecin, Poland; kamila.rachubinska@pum.edu.pl (K.R.); grochans@pum.edu.pl (E.G.); 3 Institute of Nursing and Midwifery, Medical University of Gdansk, M. Sklodowskiej-Curie Street 3a, 80-227 Gdansk, Poland; 4 Department of Basic Clinical Skills and Postgraduate Education for Nurses and Midwifes, Nicolaus Copernicus University in Torun, 1 Łukasiewicza Street, 85-821 Bydgoszcz, Poland; anna.andruszkiewicz@cm.umk.pl; 5 Department of Internal Medicine and Community Nursing, Institute of Nursing and Midwifery, Faculty of Health Sciences, Jagiellonian University-Medical College, 31-007 Krakow, Poland; ilona.kuzmicz@uj.edu.pl; 6 Medical College, Jan Kochanowski University of Kielce, 25-369 Kielce, Poland; dkoziel@ujk.edu.pl

**Keywords:** loneliness, optimism, self-efficacy, COVID-19, pandemic, student

## Abstract

(1) The COVID-19 pandemic is a global epidemic crisis situation with negative health consequences. This study aimed to determine the mediatory role of self-efficacy in correlations between dispositional optimism and loneliness (both general loneliness and social and emotional loneliness) among Polish nursing students during the COVID-19 pandemic. (2) The study involved 894 students from six Polish universities. A diagnostic survey was used as the research method, and the Scale for the Measurement of Loneliness Scale (DJGLS), Life Orientation Test (LOT-R), and the Generalized Self-Efficacy Scale (GSES) were used to collect data. (3) The mean subject age was 20.73 years (SD = 1.81). More than half (51.01%) of the respondents scored high on the GSES scale, indicating an individual’s belief in the self-efficacy in coping with difficult situations and obstacles. However, 40.60% scored low on the LOT-R scale, indicating that the respondents were pessimistic. The mediation analysis revealed that self-efficacy plays a partial mediatory role in correlations between dispositional optimism and loneliness in general, social and emotional loneliness. (4) It is important to undertake loneliness prophylactic and prevention activities among nursing students and to develop personal resources, i.e., optimism and self-efficacy, to effectively offset the effects of the COVID-19 pandemic.

## 1. Introduction

The COVID-19 pandemic, which started in the first quarter of 2020, continues to have a great impact on many aspects of human life. People’s responses during this time have varied because of the “novelty” of the situation, which manifested itself in a reduced scale of contact with other people and difficulty participating in social or professional life [1]. The extent of the situation justifies the claim that a certain part of the population experiences loneliness [2,3,4,5].

De Jong Gierveld defines loneliness as an unpleasant, unacceptable situation of lacking (the quality of) specific social relations. This applies to situations in which the number of existing relations is smaller than desired (expected), as well as to those in which the need for closeness is not satisfied [6]. It is a consequence of a cognitive evaluation of the number and quality of existing relations and the individual’s attitude towards those relations [7,8,9]. Findings of empirical analyses show that loneliness can cause many life issues. Loneliness is linked to the deterioration of social bonds, a lower frequency of interpersonal contact, a weaker sense of competence in various spheres of everyday life, a lower income, and a lower education level [10,11]. One can identify a (physical, objective) sense of social loneliness, whose characteristic features include an absence of social bonds, a lack of belonging to a community, experiencing isolation and marginalisation, and a sense of being ignored. Existential loneliness is another form, and it denotes a lack of identification with generally accepted values, life goals, and standards [12]. The third type is emotional (subjective) loneliness, which can be described as emotional solitude. It indicates a deficit of positive feelings and relations with people, especially with those who are significant to an individual. The typical features include a negative opinion of one’s social skills, a lack of acceptance of and trust in oneself and others, lowered mood, as well as negative emotions and feelings [12,13]. The research conducted to date, focusing on the consequences of restrictions imposed on the society to control the spread of the COVID-19 pandemic, has shown loneliness to be one of them [14,15]. Nearly half of the population aged 18–24 years feel a higher level of loneliness than elderly people during isolation [4,14,16,17]. The research conducted by Labrague et al. showed loneliness to dominate among students during the pandemic, whereas social support and coping behaviours were identified as factors protecting them from loneliness [18].

An increase in the SARS-CoV-2 infection rate necessitated changes in the higher education and science system. Online or hybrid teaching was introduced. Mandatory social distancing and isolation made the situation even more difficult. A study conducted among students in Poland during the second wave of the pandemic shows decreasing life satisfaction and an increasing level of stress symptoms. Negative emotions of anxiety, sadness, exhaustion, and loneliness dominated among young people [19]. Social distancing and longing for direct contact with other people were the major issues experienced by students [20]. They are worried that the pandemic will have a long-term impact on their everyday lives and their future [21].

The COVID-19 pandemic has set new challenges regarding the theoretical and clinical education of nursing students. The study findings show that the SARS-CoV-2 virus spread has caused students’ mental states to deteriorate considerably [22,23]. The findings of studies conducted by Eweid et al. showed that nursing students were the most stressed by the risk of infection and virus transmission to a relative at clinical classes during the COVID-19 pandemic [22].

Because of the specific nature of the nurse and midwife professions, they (and even nursing students) are required to be psychologically resilient, sensitive to pain and suffering, caring, and eager to help. Resistance to stress and the ability to cope in difficult situations is also important, which is why psychological factors and their functions that have an impact on professional accomplishments and success in life should be investigated. Optimism is one of an individual’s personal resources. Studying its relationship with emotional control provides knowledge of psychological strength, which helps to improve nurses’ and midwives’ functioning in their professional and familial environments. Optimism is a personality trait with a positive impact on the physical condition, well-being, success in life, and resistance to stressors [24]. The theory of dispositional optimism, developed by Scheier and Carver, is the most popular theory that deals with optimism. According to it, dispositional optimism is a personality trait that concerns an individual’s generalised expectations regarding the future [25]. This trait is a behaviour-regulating mechanism, as an individual’s expectations regarding the future have a significant impact on the method of accomplishing the life objectives and set directions for activities taken to accomplish them [26]. Schwarzer describes two types of optimism: defensive, which denotes an unrealistic opinion of the subjective chances of avoiding unpleasant events and functional, understood as high self-efficacy. Understood in this way, an optimist allows for failure but interprets it in a manner favourable to him/herself [27].

People’s responses during the pandemic and their impact on their self-efficacy are interesting because of the “novelty” of the situation, which manifests itself in a reduced scale of interpersonal contacts and difficulty participating in social and professional life [1]. The concept of self-efficacy has become of key importance in many psychology areas, especially in health psychology, where it constitutes the main determinant of changing one’s health behaviour [28]. A higher self-efficacy boosts an individual’s motivation for action and results in his/her better accomplishments [29,30]. Self-efficacy is a belief in one’s ability to organise and implement such actions that will be necessary to overcome potential future obstacles. It is an individual’s specific opinion on his/her competence and ability to perform different tasks in a given field [31,32,33]. Bandura identifies four main factors that have an impact on an individual’s self-efficacy. These include:—actual accomplishments—providing information about our strengths and weaknesses, capabilities, talents, and limitations—vicarious experience—this is the phenomenon of observing other people similar to us, who attain their goals by making continuous efforts—persuasion—information from other people on what kind of people we are and what we can achieve—emotional stimulation, which manifests itself in the physiological condition of the organism, associated with self-efficacy in a specific situation [34].

These considerations show that identifying two types of personal resources: optimism and self-efficacy, which are clearly present in the literature on the subject, helps one conduct an in-depth and precise investigation of links concerning the social and emotional loneliness of nursing students.

This study aimed to determine the mediatory role of self-efficacy in correlations between dispositional optimism and loneliness, understood both as general loneliness as well as social and emotional loneliness among nursing students in Poland during the COVID-19 pandemic.

## 2. Materials and Methods

### 2.1. Settings and Design

The study was conducted between 20 March and 15 December 2021 among a group of nursing students in a first-cycle (bachelor’s degree) full-time programme. The students’ age being under 30 years was the enrolment criterion, and the participants had to give their informed consent for participation in the study. Those who failed to give such consent were excluded from the study. The survey was conducted at the University of Warmia and Mazury in Olsztyn (*n* = 175; 19.57%), Medical University of Gdańsk (*n* = 143; 16.00%), Jagiellonian University in Kraków (*n* = 132; 14.77%), Nicolaus Copernicus University in Toruń, Collegium Medicum in Bydgoszcz (*n* = 171; 19.24%), Jan Kochanowski University of Kielce (*n* = 57; 6.38%) and the Pomeranian Medical University in Szczecin (*n* = 215; 24.05%). Having obtained the deans’ consent, researchers at each of the universities conducted the survey while maintaining the epidemic safety guidelines in direct contact with the students. The students were informed about the study objective, and they had an opportunity to ask questions and receive the answers. After they gave their consent to participate in the study, they received survey questionnaire packages. Altogether 975 questionnaire sets were distributed. The respondents could withdraw from the study at any moment without giving a reason. It took approximately 15 min to complete the questionnaire. After the data were collected and incomplete questionnaires eliminated, 894 packages (i.e., 91.69%) were taken for further statistical analysis. This study is part of a larger research project, which was given a favourable opinion (No. 3/2021) by the Senate Scientific Research Ethics Committee at the Olsztyn University in Olsztyn.

### 2.2. Participants

The study included 894 nursing students, with females (822; 91.95%) accounting for a large majority of the respondents. The respondents’ mean age was 20.73 years (SD = 1.81). There were 397 (44.41%) first-year students, 289 (32.33%) second-year students, and 208 (23.27%) third-year students. The students usually lived with family or with someone close (69.46%), they usually spent 6.08 h working online, 27.29% of the study subjects restricted their physical activity during the COVID-19 pandemic, and walking was the most common form of activity (47.48%). They usually had 3–4 meals daily (74.05%), and nearly half of them (40.27%) claimed their social contact was considerably restricted during the COVID-19 pandemic. Nearly all the study subjects (97.32%) described their health status as very good or good (Table 1).

### 2.3. Research Instruments

The diagnostic survey method was applied and three standardised research tools, adapted to the Polish conditions, were used to collect empirical data:

- The Loneliness Measurement Scale (De Jong Gierveld Loneliness Scale—DJGLS) developed by J. de Jong-Gierveld and F. Kamphuis, a Polish adaptation developed by Grygiel et al. [35,36];
- The Life Orientation Test-Revised (LOT-R) developed by Michael F. Scheier, Charles S. Carver, Michael W. Bridges, a Polish adaptation developed by R. Poprawa, Z. Juczyński [37];
- The Generalised Self-Efficacy Scale (GSES) developed by R. Schwarzer, M. Jerusalem, a Polish adaptation developed by R. Schwarzer, M. Jerusalem, Z. Juczyński [37].

The study group was characterised by the use of an original questionnaire developed by the authors. It contained questions about basic sociodemographic data and on selected lifestyle-related issues.

#### 2.3.1. De Jong Gierveld Loneliness Scale (DJGLS)

The De Jong Gierveld Loneliness Scale (DJGLS; developed by J. de Jong-Gierveld and F. Kamphuis, in a Polish adaptation developed by Grygiel et al.) is basically one-dimensional, and it measures generalised loneliness. It is a partly balanced tool, consisting of five positive items (i.e., emotional loneliness), measuring satisfaction with interpersonal relations, and six negative ones describing a lack of social contact (i.e., social loneliness). The respondents marked the level of acceptance of each statement on a five-item scale, from “definitely yes” to “definitely not”. After the “negative” elements are transposed, a higher total score indicates a more intense feeling of loneliness. The scale is highly reliable and homogeneous: the internal stability index (Cronbach alpha) is 0.89, inter-item correlation r = 0.42, and Loevinger’s H coefficient of homogeneity is 0.47, indicating a medium relationship between the forming the scale. In this study, the internal consistency of DJGLS was Cronbach alpha = 0.87 for emotional loneliness, and Cronbach alpha = 0.86 for social loneliness [35,36].

#### 2.3.2. Life Orientation Test—Revised (LOT-R)

The Life Orientation Test—Revised (LOT-R) in a Polish adaptation by Poprawa and Juszczyński is used to measure dispositional optimism. It contains ten statements—six of them with a diagnostic value for dispositional optimism. Three statements are positive, and three are negative. The respondents use a five-point scale to evaluate to what extent a statement applies to them. The overall score lies within the interval between 0 and 24 points. The higher the score, the higher the level of optimism. After being converted to standardised units on the sten scale, a raw score helps to assess the intensity of dispositional optimism. The scores of 1–4 sten are indicative of proneness to pessimism, whereas those between 7 and 10 indicate an optimistic attitude. The LOT-R scale has good psychometric properties. For the original version, Cronbach’s alpha coefficient is 0.78, while “test-retest” studies conducted in four different groups of students, studied at 4-, 12-, 24- and 28-months intervals, yielded correlation coefficients of 0.68, 0.60, 0.56, and 0.79, respectively [37].

#### 2.3.3. Generalised Self-Efficacy Scale (GSES)

The GSES scale developed by Schwarzer et al. measures the strength of an individual’s general conviction regarding his/her efficacy in coping with difficult situations and adversities. It consists of ten statements included in one factor. The study subjects showed their acceptance of a statement on a four-degree scale, from “no” to “yes”. The total score gives a general self-efficacy index, which lies between 10 and 40 points. The higher the score, the higher the self-efficacy. After being converted to standardised units, the overall index was interpreted according to the properties of the sten scale. The scores of 1–4 sten were regarded as low, 5–6—average and 7–10—high. The GSES scale has good psychometric properties. The Cronbach alpha is 0.85, and the scale reliability evaluated by the test–retest method was 0.78 (after five weeks) [37].

### 2.4. Statistical Analysis

A statistical analysis of the data was performed with the Polish version of STATISTICA 13 (TIBCO, Palo Alto, CA, USA). The values of the measurable parameters under study were shown with the mean, median, standard deviation, minimum and maximum, and the confidence interval of the mean, and the immeasurable ones—with the sample size and the percentage. Pearson correlation (r) was used to examine the relationship between the variables [38]. The mediatory effects were measured by the methodology developed by R.M. Baron and D.A. Kenny [39]. The Sobel test was used to verify the statistical significance of the mediation analysis model [40]. The significance level of *p* < 0.05 was adopted, which is indicative of statistically significant correlations. The study meets the criteria for a cross-sectional study [41].

## 3. Results

### 3.1. Analysis of Variables

The study was conducted on a sample of 894 first-degree nursing students at six Polish universities. Table 2 presents descriptive statistics of the data obtained in the study group.

**Figure 1:**
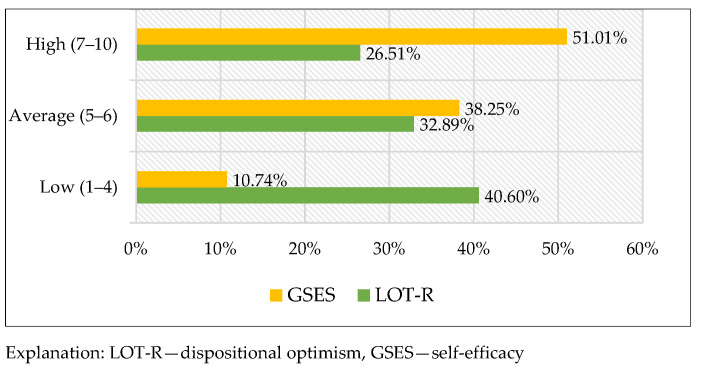
The structure of the results for self-efficacy and dispositional optimism on the sten scale.

The raw score was converted to standardised units on the sten scale to assess the intensity of dispositional optimism. The structure of the results shows a high percentage (40.60%) of students with low scores indicative of proneness to pessimism. However, high scores, which show an optimistic attitude, were achieved by 26.51% of the respondents. The average intensity of dispositional optimism was determined in 32.89% of the students (Figure 1).

The generalised self-efficacy measurement showed high scores on the sten scale for over half of the respondents (51.01%), which is indicative of their general conviction regarding their efficacy in coping with difficult situations and adversities. Average scores were noted for over one third, whereas low scores were noted for a small percentage of the respondents (10.74%) (Figure 1).

### 3.2. Mediation Analysis

A mediation analysis was performed in order to check whether self-efficacy (intervening variable) is a significant mediator in the correlation between dispositional optimism (independent variable) and loneliness (dependent variable), understood both as general loneliness as well as social and emotional loneliness. The correlations between the variables under analysis were examined at the first stage. The results of the analyses revealed statistically significant correlations between the loneliness categories on the one hand and dispositional optimism and self-efficacy on the other. The highest, negative correlation coefficient was determined for self-efficacy (r = −0.341; *p* < 0.0001). The analysis also revealed a significant, positive correlation between dispositional optimism and self-efficacy (r = 0.486; *p* < 0.0001) (Table 3).

The classic Baron and Kenny mediation model was applied in the next stage of statistical analyses. The following assumptions were adopted to test the mediation correlations:

- path c = a direct correlation between an independent variable and a dependent variable,
- path a = a correlation between an independent variable and a mediator,
- path b = a correlation between a mediator and a dependent variable (with the independent variable control),
- path c’ = a correlation between an independent variable and a dependent variable (with a mediator control).

The existence of significant correlations (*p* < 0.05) between dispositional optimism and self-efficacy (path a) and between self-efficacy and loneliness in three categories (path b) was assumed to identify a mediation effect.

#### 3.2.1. Correlation between Dispositional Optimism and Global Loneliness with a Mediatory Impact of Self-Efficacy

**Figure 2:**
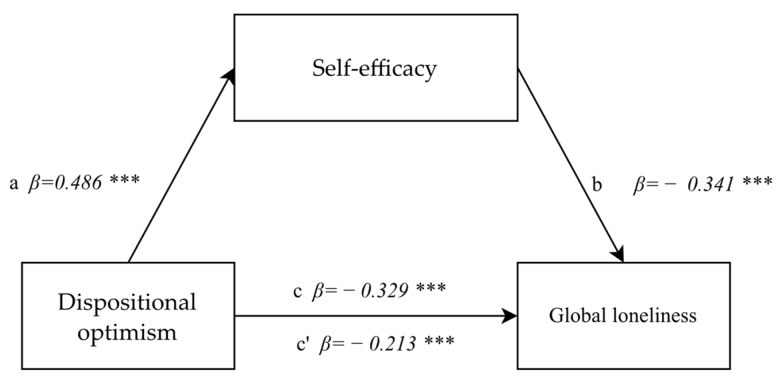
The model of a mediatory role of self-efficacy between dispositional optimism and global loneliness. Statistically significant: *** *p* < 0.001.

The mediatory impact of self-efficacy in the correlation between dispositional optimism and global loneliness was tested at a later stage of statistical analyses. First, the correlation between dispositional optimism and global loneliness was tested and found to be significant as required by the Baron and Kenney’s procedure for testing mediation effects (F(1.892) = 108.39; *p* < 0.0001; *β* = −0.329; *p* < 0.0001) and it was indicative of a negative correlation. A statistically significant, positive correlation of an average power between dispositional optimism and self-efficacy (F(1.892) = 275.15; *p* < 0.0001, *β* = 0.486; *p* < 0.0001) was confirmed in the second step of the analysis. When the mediator self-efficacy was introduced in the third step, the correlation between dispositional optimism and global loneliness decreased, but it remained statistically significant (*β* = −0.213; *p* < 0.0001), but the mediator remained strongly negatively linked with global loneliness (F(1.892) = 117.80; *p* < 0.0001, *β* = −0.341; *p* < 0.0001). The analysis demonstrated partial mediation effects, confirmed by the Sobel test result (z = −9.19; *p* < 0.0001) (Figure 2).

#### 3.2.2. Correlation between Dispositional Optimism and Social Loneliness with a Mediatory Impact of Self-Efficacy

**Figure 3:**
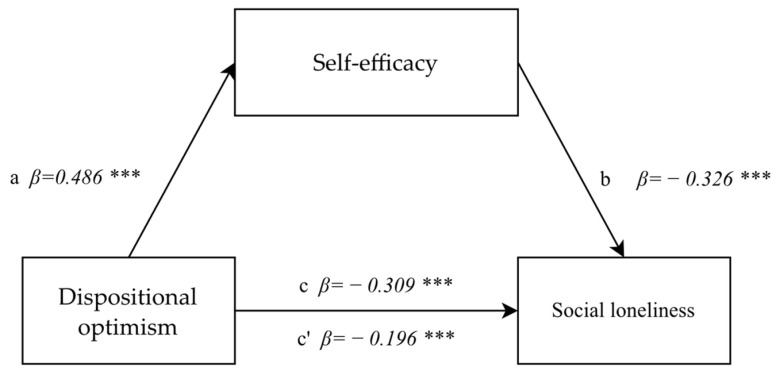
The model of a mediatory role of self-efficacy between dispositional optimism and social loneliness. Explanation: Statistically significant: *** *p* < 0.001.

The mediatory impact of self-efficacy in correlation between dispositional optimism and social loneliness was tested at a later stage of statistical analyses. First, a correlation between dispositional optimism and social loneliness was tested and found to be significant (F(1.892) = 94.020; *p* < 0.0001, *β* = −0.309; *p* < 0.0001). The correlation between dispositional optimism and self-efficacy was then tested in the second step. The correlation proved to be (F(1.892) = 275.15; *p* < 0.0001; *β* = 0.486; *p* < 0.0001). This means that the higher the dispositional optimism level, the higher the self-efficacy. A mediator was introduced to the model in the third step, and the significance of the mediation model was evaluated. The magnitude of the correlation between dispositional optimism and social loneliness decreased after the mediator self-efficacy was introduced, but it remained significant (*β* = 324 −0.196; *p* < 0.0001), and the mediator remained negatively linked to social loneliness (F(1.892) = 106.15; *p* < 0.0001, *β* = −0.326; *p* < 0.0001). The analysis demonstrated partial mediation effects, confirmed by the Sobel test result (z = −8.90; *p* < 0.0001) (Figure 3).

#### 3.2.3. The Correlation between Dispositional Optimism and Emotional Loneliness with a Mediatory Impact of Self-Efficacy

**Figure 4:**
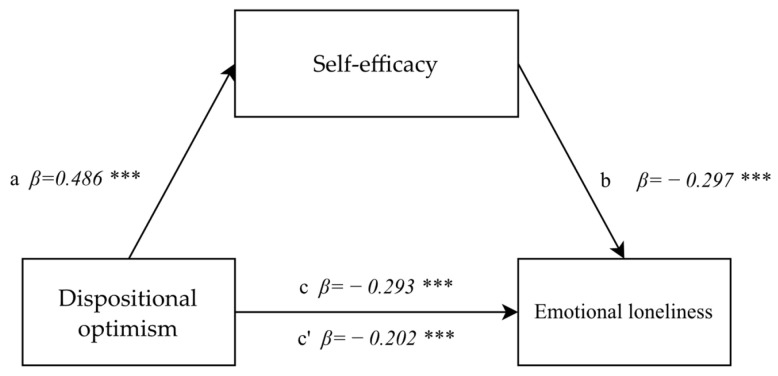
The model of a mediatory role of self-efficacy between dispositional optimism and emotional loneliness. Explanation: Statistically significant: *** *p* < 0.001.

The mediatory function of self-efficacy in the correlation between dispositional optimism and emotional loneliness was tested at the last stage of the statistical analysis. The analyses performed at the first step found the correlation between dispositional optimism and emotional loneliness to be significant (F(1.892) = 83.911; *p* < 0.0001, *β* = −0.293; *p* < 0.0001) and it was indicative of a negative low correlation. In the second step, the correlation between dispositional optimism and the mediator (self-efficacy) was tested and found to be significant and positive (F(1.892) = 275.15; *p* < 0.0001, 342 *β* = 0.486; *p* < 0.0001). After the mediator, self-efficacy, was introduced in the third step, the correlation between dispositional optimism and emotional loneliness decreased slightly although remaining significant (*β* = −0.202; *p* < 0.0001). The mediator was weakly and negatively correlated to emotional loneliness (F(1.892) = 86.055; *p* < 0.0001; *β* = −0.297; *p* < 0.0001). The analysis demonstrated partial mediation effects, confirmed by the Sobel test result (z = −8.3; *p* < 0.0001) (Figure 4).

## 4. Discussion

There have been many study reports in the literature on the subject confirming that health issues among students during the COVID-19 pandemic include depression, fear, anxiety, stress, sleep disorders, and loneliness [42,43,44,45].

This phenomenon is seen to be the most widespread among young adults aged 18–25 years, which corresponds to the time of studying at university [4].

Cacioppo et al. found that 20–48% of the young adults and adolescents in their study reported loneliness, often high in severity [3]. These groups may have the highest percentages among all population groups [5,46,47]. For nursing students, the restriction or blocking of their interpersonal contact during the COVID-19 pandemic may have played a significant role in developing or increasing feelings of loneliness.

Moreover, cross-cultural comparisons of social relationships in relation to the concept of individualism vs. collectivism showed that loneliness increased with individualism, decreased with age, and was higher in men than in women [17]. Morrish et al. argued for a bidirectional relationship between loneliness and unemployment by analysing longitudinal data from a representative sample of the working-age population. They argued that reducing loneliness may alleviate distress from unemployment, and employment may reduce loneliness, which in turn may have positive effects on health and quality of life. To combat loneliness, they suggested that employers and governments need to make additional supportive services more widely available [48].

Diehl et al. found that emotional loneliness was more widespread than social loneliness among the students, and both variables were correlated positively to a sense of depression and anxiety [49]; in addition to loneliness, they also point to other health consequences due to the spread of COVID-19.

For example, Savarese showed that students experienced a high level of stress and anxiety and concentration and sleep disorders during the pandemic [42]. Długosz provided much information on the changes in student functioning caused by the pandemic outbreak. The findings showed that the frequency of depressive symptoms, dizziness, and stress in general increased during the pandemic [50]. Students started to take actions aimed at stress reduction and a large majority of them became more active, which helped them divert their attention away from the current situation. A considerable part of the employ rationalisation, i.e., they seek positive aspects of the situation and focus on positive aspects of their lives [50]. They spend more time on the Internet, which is not only because they have more free time, but because the way that they work or learn has changed. The available study findings show that people try to adapt to the situation at hand during the pandemic and reduce the level of discomfort by using the resources at their disposal [50,51]. Restrictions of contact with other people, especially personal ones, may provoke a range of emotions, such as anger, anxiety, and disappointment. Contact with other people or an ability to communicate is an important element of social roles, and it favours development and well-being [52].

It is important to possess personal resources to cope with difficult situations, and the COVID-19 pandemic certainly is one. The authors of this study see optimism as playing a significant role in better physical and mental adaptation to any pandemic-related restrictions. The study findings show that nursing students in Poland have achieved a mean dispositional optimism score of 13.35 points on a scale from 0 to 24, which is slightly lower than in the normalisation study (14.55) conducted on a group of healthy people [33]. Students in the study by Sławska also achieved a higher mean dispositional optimism index (15.18) than in this study [53]. It is worrying that 40.60% of the nursing students taking part in the study demonstrated a low level of dispositional optimism, which is indicative of a tendency for pessimism. Only a quarter of the students (participants) exhibited a highly optimistic attitude.

Sławska argues that the students’ marks and learning progress were significantly correlated to the dispositional optimism level, which was high in students with better marks, who improved their learning achievements. A high dispositional optimism level had a considerable beneficial impact on nursing students’ learning effectiveness [54]. The findings of Zhao’s study also showed an optimistic attitude toward improving learning achievements. This study demonstrated that optimism plays an important role in self-education with various levels of loneliness and attentiveness [55]. Krif stressed the importance of optimism as a mechanism with the potential to increase the level of emotional regulation and decrease depression and anxiety [54]. Its findings showed that those students who demonstrated a higher level of optimism were more engaged in studying, which resulted in a lower level of depressiveness. These findings stressed the potentially protective role of optimism for students during the COVID-19 pandemic. These are consistent with earlier findings, according to which optimism was positively correlated to the students’ mental health [56,57]. Such effects can be attributed to the fact that optimism is associated with higher perceived social support and higher use of active coping strategies, including the seeking of social support [58].

The mediation model used in this study demonstrated a negative correlation between dispositional optimism on the one hand and global loneliness, as well as social and emotional loneliness on the other. This means that dispositional optimism plays a modifying function in nursing students’ various actions and types of behaviour.

According to Mróz, the frequency of contact with other people is important both for loneliness and for dispositional optimism [1]. This probably results from the fact that such contacts determine an individual’s belonging to various social groups and are determinants of the social networks to which such an individual belongs.

The authors of this study point out that students can take specific actions during the pandemic and change adverse behaviours into beneficial behaviours if they are convinced that they have the appropriate skills for this. Self-efficacy can help them to do it.

The nursing students in this study achieved mean self-efficacy scores of 29.39 points on a scale from 10 to 40, whereas the mean score was a little lower in the normalisation group (27.32) [37]. The mean score of 28.63 was obtained in a comparative study on a sample of 12,840 participants, covering 14 different cultures [38]. The generalised self-efficacy measurement showed high scores on the sten scale for over half of the respondents (51.01%), which is indicative of their general conviction regarding their efficacy in coping with difficult situations and adversities, whereas low scores were observed in a small percentage (10.74%) of the study participants.

Dispositional optimism correlated positively with self-efficacy in the mediation model presented here. Self-efficacy is of key importance in turning intention into action. There are multiple factors which can amplify self-efficacy and have a direct or indirect impact on an individual’s health [59,60]. For example, a study by Xiao among medical personnel during the COVID-19 pandemic showed that social support provided to medical personnel reduced anxiety and stress and increased self-efficacy [59]. Social support contributes to the improvement of self-efficacy, resulting in better understanding, higher respect, encouragement, courage, and satisfaction with one’s professional achievements [60]. Self-efficacy results in higher self-confidence in doing one’s job and combined with social support, reduces the suffering caused by loneliness and helps medical professionals to be more optimistic, which improves the mechanisms of coping with stress. Self-efficacy also improves concentration and self-control [61]. Although a majority of medical personnel feel pressured at work, individuals with high self-efficacy are able to control their emotions. Anxiety has been shown to increase susceptibility to pressure at work and to have a negative impact on self-efficacy, as it reduces positive behaviours and initiative [62,63,64]. In their study, Han et al. revealed that stress caused by e-learning has a negative impact on self-efficacy in members of the academic community [65]. Earlier studies showed that the COVID-19 pandemic had had a negative impact on self-efficacy, and a majority of students in the online teaching regime had experienced negative emotions, including anxiety and stress [66,67,68,69,70]. In particular, a reduction in interpersonal relations in the online teaching environment can strengthen the existing feeling of being marginalised in students and make them develop a new feeling of isolation [71]. Similar findings were obtained by Rohmani et al., who demonstrated a significant correlation between self-efficacy among the academic community and burnout among first-year nursing students during online teaching [72]. Students with low self-efficacy in learning experienced severe burnout more frequently and vice versa. Academic self-efficacy is associated with an individual’s experience and maturity. Students with low or moderate self-efficacy in learning will be able to discover their skills and make their own decisions less frequently [72].

In a mediation analysis, the authors of the present study demonstrated that self-efficacy was a partial mediator that reduced the correlation between dispositional optimism and loneliness. An analysis of the literature shows that no scientific research on optimism or self-efficacy in association with loneliness among nursing students during the COVID-19 pandemic has been conducted in Poland recently.

The findings can be regarded as reflecting nursing students’ personal resources, which are helpful in coping with adversity and support the optimum learning process. This claim was confirmed by the findings of Gandhi, which demonstrated a positive correlation between self-efficacy and optimism [73]. A high level of optimism has been demonstrated to contribute to a positive correlation with adaptation, it can improve an individual’s cognitive and emotional functioning when he/she is facing a challenge, or it can reduce the psychological stress level. Individuals with higher self-efficacy, optimism, and attentiveness are better mentally prepared to function during the pandemic [73]. Said et al. confirmed that self-efficacy, self-esteem and dispositional optimism could be regarded as predictors for the evaluation of nurses’ psychological preparedness for disasters [74]. Many authors have pointed out that if self-efficacy in coping with depression is high, individuals tend to think in a manner which facilitates overcoming obstacles, and they follow positive coping strategies. As a consequence, they experience negative emotions less frequently [75,76,77]. If self-efficacy in expressing positive emotions is high, individuals tend to think positively and experience self-acceptance and self-worth [78]. Such a positive thinking style favours positive expectations regarding the future and can reduce the probability of negative emotions [79].

### Limitations and Implications Regarding Professional Practice

The authors of this study suggest that there is a need to intensify actions aimed at exploring students’ needs during the COVID-19 pandemic. It is also necessary to take institutional prophylactic actions to prevent loneliness among young people during and after the COVID-19 pandemic. The major strengths of this study include a rather large sample and nursing students enrolled from several academic centres in various parts of Poland, which can be regarded as a representative sample for the country. Moreover, open-ended questions in the questionnaire, apart from the standardised tools, enriched the data presented here.

The study also has its limitations, in that, the students who also studied a second course during the research or who had financial issues were not excluded. Another limitation is the absence of data on support received by students from institutions or from their families or personal health issues.

Despite these limitations, this study provided important findings, and it can be a starting point for more extensive research on how young people respond to the restriction of social contact and on the importance of relations with others in the aspect of loneliness, life optimism and the role of self-efficacy.

This study suggests that academic teachers should consider the need to develop and implement programmes aimed at improving students’ attentiveness while at the same time decreasing their loneliness and encouraging students to adopt an optimistic attitude, increasing emotional adaptation and self-efficacy. By looking after their students, academic teachers can teach them to care about lonely patients. Developing a supportive organisational culture in the educational context may be beneficial and could be an object of further research.

## 5. Conclusions

In the mediation model adopted, self-efficacy was shown to mediate the correlation between dispositional optimism and loneliness both in terms of general loneliness and social and emotional loneliness, playing an important role as a partial mediator.

Self-efficacy was only a partial mediator that reduced the correlation between optimism and loneliness.

When controlling for self-efficacy, higher scores on optimism were associated with lower scores for loneliness.

It is important to undertake activities related to loneliness prophylactic and prevention activities among nursing students and to develop personal resources, i.e., optimism and self-efficacy, in order effectively offset the effects of the COVID-19 pandemic effectively.

## Figures and Tables

**Table 1:** Sociodemographic characteristics of the study group.

Variables		Total N = 894	
		Number	%
Gender	female	822	91.95
	male	72	8.05
Study year	first	397	44.41
	second	289	32.33
	third	208	23.27
Age (years) M = 20.73; SD = 1.81	≤20	481	53.80
	21–22	319	35.68
	≥23	94	10.51
Place and form of residence	with family/someone close	621	69.46
	on their own	273	30.54
Number of hours spent working on a computer M = 6.08; SD = 3.19	≤5	433	48.43
	6–9	302	33.78
	≥10	159	17.79
Number of consumed meals per day M = 3.48; SD = 0.87	1–2	104	11.63
	3	382	42.73
	4	280	31.32
	≥5	128	14.32
Restriction of physical activity during the pandemic	no	211	23.60
	yes, to a small extent	161	18.01
	yes, to a medium extent	278	31.10
	yes, to a considerable extent	244	27.29
Subjective health status assessment during the pandemic	bad	24	2.68
	good/ average	613	68.57
	very good	257	28.75
Restriction of social contacts during the pandemic	very high	141	15.77
	considerable	360	40.27
	medium/ average	229	25.62
	to a small extent	164	18.34

Explanations: N—number of subjects.

**Table 2:** Descriptive statistics of the variables under analysis.

Variables	N = 894				
	M	95% CI	Me	Min.–Max.	SD
LOT-R	13.35	13.05–13.65	14	0–24	4.60
GSES	29.39	29.11–29.67	30	14–40	4.24
SS	14.53	14.16–14.90	14	4–30	5.68
SE	11.14	10.86–11.42	11	4–25	4.23
GPS	25.67	25.08–26.27	25	9–51	9.10

Explanation: N—sample size, M—arithmetic mean, 95% CI—confidence interval of the mean, Me—median, Min.—minimum, Max.—maximum, SD—standard deviation, LOT-R—dispositional optimism, GSES—self-efficacy, SS—social loneliness, SE—emotional loneliness, GPS—global loneliness.

**Table 3:** Pearson correlation (r) coefficient matrix between the variables under analysis.

	Variables	1.	2.	3.	4.
1.	LOT-R	-			
2.	GSES	0.486 ***	-		
3.	SS	−0.309 ***	−0.326 ***	-	
4.	SE	−0.293 ***	−0.297 ***	0.678 ***	-
5.	GPS	−0.329 ***	−0.341 ***	0.940 ***	0.888 ***

Statistically significant: *** *p* < 0.001. Explanation: LOT-R—dispositional optimism, GSES—self-efficacy, SS—social loneliness, SE—emotional loneliness, GPS—global loneliness.

## Data Availability

The data presented in this study are available on request from the first author.
